# Effect of Humic Acid Addition on Buffering Capacity and Nutrient Storage Capacity of Soilless Substrates

**DOI:** 10.3389/fpls.2021.644229

**Published:** 2021-07-26

**Authors:** Jingcheng Xu, Esraa Mohamed, Qiang Li, Tao Lu, Hongjun Yu, Weijie Jiang

**Affiliations:** ^1^Key Laboratory of Horticultural Crops Genetic Improvement (Ministry of Agriculture), Institute of Vegetables and Flowers, Chinese Academy of Agricultural Sciences, Beijing, China; ^2^Taizhou Academy of Agricultural Sciences, Taizhou, China

**Keywords:** humic acid, substrate properties, cucumber, pH buffering capacity, storage capacity

## Abstract

Excessive application of fertilizers has become a major issue in croplands of intensive agricultural systems in China, resulting in severe non-point source pollution; thus, reduction in the use of chemical fertilizers has received significant attention. Improving the nutrient storage capacity of soils or substrates is an effective approach for solving this problem. Humic acids (HA) are excellent soil conditioners. Thus, in the present study, their ability to improve the physico-chemical properties of three substrates with different textures was evaluated. HA treatments included 1% HA root application in three different types of substrates, including pure sand, pure cocopeat, and a mixture of sand:cocopeat (1:1, v/v) and their relative controls. We examined the morphological parameters of cucumber seedlings as well as pH buffering capacity (pHBC), total organic carbon (TOC), organic matter (OM), cation exchange capacity (CEC), and nutrient storage capacity of the three substrates. The results show that HA application improved the morphological parameters of cucumber seedlings (plant height, stem diameter, and biomass) in pure cocopeat and cocopeat-sand mixture treatments. On the contrary, HA addition had harmful effects on the cucumber seedlings cultivated in sand due to the low pHBC of sand. The seedlings cultivated in pure cocopeat showed the best morphological parameter performances among the seedlings grown in the three substrates. Furthermore, pHBC, TOC, OM, and CEC were enhanced by HA application. Incorporation of HA improved ammonium (NH_4_^+^) and potassium (K^+^) storage capacity while decreasing phosphorus (P) storage. Pure cocopeat had the highest pHBC, TOC, OM, CEC, and nutrient storage capacity among the three substrates. In conclusion, mixing 1% HA into substrates promoted cucumber growth, improved substrate properties, and enhanced fertilizer use efficiency. Pure cocopeat is a suitable substrate for cucumber cultivation, and mixing cocopeat with sand amends the substrate properties and consequently improves plant growth.

## Introduction

Chemical fertilizers are one of the most important factors in the agricultural production of modern civilization, and they can dramatically enhance crop yield. In China, the unprecedented population boom is threatening food production self-sufficiency (Wei et al., 2015). In an attempt to make the most of the limited productive arable land and achieve high food production sustainably, farmers tend to cultivate high-yielding varieties which have a high demand for nutrients. Therefore, substantial quantities of synthetic fertilizers are chronically input into fields (Cai et al., 2002; Chen et al., 2004; Cui et al., 2008; He et al., 2009; Yan et al., 2012). However, irrational agricultural practices lead to low fertilizer use efficiency and severe agricultural non-point source pollution (Sun et al., 2012; Fan et al., 2014). Compared with developed countries, China lost more nutrients in agricultural production, and fertilizer use efficiency of nitrogen (N), phosphorus (P), and potassium (K) was less than 35, 20, and 50%, respectively (Li et al., 2010); thus, taking a deep look into the improvement of fertilizer use efficiency is of great significance and necessity.

In general, after fertilization, some nutrients are absorbed by the soil, and others are lost, such as leaching and volatilization loss (Ahmad et al., 1982; Dong et al., 2009). The storage capacity of nutrients (mainly N, P, and K) is considered a crucial index of soil productivity evaluation. Nutrient absorption and release play a vital role in fertilizer use efficiency as they affect soil nutrient supplies, which are important for crop nutrient uptake (Anderson and Magdoff, 2005; Simonsson et al., 2009; Nieder et al., 2011). With the current concerns regarding environmental protection and the demand for production of more food with less fertilizer application, the focus is directed toward enhancing soil nutrient storage capacity, which could be a way to improve fertilizer use efficiency to minimize fertilizer loss and leaching into the environment.

Humic acids (HA) are a new type of biostimulant that have become popular in recent years and are mainly derived from the decomposition of animal and plant remains by microbes under aerobic and anaerobic conditions and during various geochemical processes (Dogan et al., 2014). HA perform various functions in agricultural production; for instance, HA could be used as growth regulators to alter hormone levels in plants and to alleviate the deleterious effects of abiotic stress on plants (Mora et al., 2012; Bijanzadeh et al., 2019). A previous study shows that soil properties, such as aggregation, aeration, and permeability, could be greatly improved by HA application in saline-sodic soils (Nan et al., 2016). K fixation was significantly reduced by incorporation of HA in brown soils (Liang et al., 2005; Lan et al., 2016). In addition, previous studies demonstrate that the addition of HA significantly improved P availability in inceptisol (Cimrin and Yilmaz, 2005). In general, previous studies mainly focus on the effects of HA application on natural soil types with similar physico-chemical properties. For example, the bulk density of saline-sodic soils and brown soils is about 1.40 g⋅cm^–3^, and the organic matter (OM) content of saline-sodic soils, brown soils, and inceptisol is about 10 g⋅kg^–1^. Soilless substrates have proper pH value, lighter bulk density and no soil-borne disease, which can replace soil to provide a good growth environment for plants (Vaughn et al., 2011; Banitalebi et al., 2021). Nowadays, more and more farmers are inclined to use soilless substrates for vegetable cultivation in protected fields (Gao et al., 2018). Limited studies have been carried out on the effects of HA application on soilless substrates with significantly different bulk density, porosity, OM, pH buffering capacity (pHBC), and nutrient storage capacity. Cocopeat is an organic soilless substrate made of coir, which is suitable for use as a growing substrate due to its excellent physical properties, such as high water-holding capacity and porosity (Abad et al., 2005). Cucumber is widely cultivated in China on account of its delicious taste, short nutritional cycle and high economic benefits (Zhao et al., 2019). According to the results of preliminary experiments, the addition of HA to cocopeat at a rate of 1% (HA:water, w/w) significantly improved cucumber yield under 15% fertilizer reduction (unpublished). Seedling quality has a significant effect on the yield of plants (Markovic et al., 1997). Thus, in the present study, our primary aim was to investigate the effects of 1% HA application to substrates with significantly different physico-chemical properties on cucumber seedling growth and substrate properties, especially on the pHBC and nutrient storage capacity.

## Materials and Methods

### Growth Conditions and Experimental Materials

The experiment was conducted in a glasshouse at the Soilless Culture Department, Institute of Vegetables and Flowers (IVF), Chinese Academy of Agricultural Sciences (CAAS), Beijing, China, in the period from February to April 2019. Cucumber (*Cucumis sativus* L.) (cv. Zhongnong No. 26) was selected as the plant material. Cucumber seedlings were kept in a glasshouse under natural light intensity and a natural photoperiod. Cocopeat and sand were provided by the Beijing Yinong Agricultural Technology Company (Beijing, China). Characteristics of the three different substrates used in the study are shown in Table 1. Humic acid (pH: 5.74, fulvic acid: 43.94%, organic matter: 47.3%, total N: 5.98%, total K: 2.25%, total P: 0.04%, SiO_2_: 0.18%) was obtained from Shandong Quanlinjiayou Humic Acid Technology Company (Shandong, China) and was extracted from wheat straw, and there is no hormone in this humic acid.

**TABLE 1:** Basic physico-chemical properties of three different substrates.

Substrates	pH	Total N (g kg^–1^)	Total P (g kg^–1^)	Total K (g kg^–1^)	Bulk density (g cm^–3^)	Total porosity (%)	Water-holding porosity (%)	Air-filled porosity (%)
Pure sand	8.17	0.47	0.02	0.24	1.34	36.57	9.53	27.04
Pure cocopeat	6.50	6.35	0.60	8.28	0.13	76.66	57.96	18.70
Mixture of sand and cocopeat (1:1, v/v)	7.00	3.19	0.24	4.17	0.52	55.29	33.22	22.07

### Experimental Design

The trial consisted of two treatments [no HA application and 1% HA (HA:water, w/w) root application], using three types of substrates: pure sand, pure cocopeat, and a mixture of sand:cocopeat (1:1, v/v), for a total of six treatment combinations [T1: pure sand without HA, T2: pure sand with 1% HA, T3: pure cocopeat without HA, T4: pure cocopeat with 1% HA, T5: mixture of sand: cocopeat (1:1, v/v) without HA, T6: mixture of sand: cocopeat (1:1, v/v) with 1% HA]. The treatments were organized in a randomized complete block design with three replicates for each treatment, and each treatment included 18 plants. The addition rate of HA, 1% (HA:water, w/w), was determined according to previous experiments (unpublished). Cucumber seeds were germinated and sown in the trays filled with peat until the seedlings developed three true leaves. Before transplanting, HA was thoroughly mixed with different substrates [pure sand, pure cocopeat, and a mixture of sand:cocopeat (1:1, v/v)]. The seedlings were then transplanted into plastic pots containing 1.2 L of substrate [T1: pure sand without HA, T2: pure sand with 1% HA, T3: pure cocopeat without HA, T4: pure cocopeat with 1% HA, T5: mixture of sand: cocopeat (1:1, v/v) without HA, T6: mixture of sand: cocopeat (1:1, v/v) with 1% HA]. The same management practices were applied across all treatments. The nutrient solution used in the experiment was a special Yamazaki cucumber cultivation formula as shown in Table 2. The pots were watered with this nutrient solution once or twice a week, depending on substrate humidity, and the same quantity of nutrient solution was applied to all treatments.

**TABLE 2:** Composition of nutrient solution for cucumber proposed by Yamazaki.

Macronutrients	Final concentration (mg/L)	Micronutrients	Final concentration (mg/L)
KNO_3_	607	H_3_BO_3_	2.86
Ca(NO_3_)_2_⋅4H_2_O	826	MnSO_4_⋅4H_2_O	2.13
NH_4_H_2_PO_4_	115	ZnSO_4_⋅7H_2_O	0.22
MgSO_4_⋅7H_2_O	483	CuSO_4_⋅5H_2_O	0.08
		(NH_4_)_6_Mo_7_O_24_⋅4H_2_O	0.02
		Na-Fe-EDTA	30

### Morphological Measurements

At the end of the experiment, 18 plants were randomly sampled from each treatment to measure plant height and stem diameter using a ruler and an electronic vernier caliper, respectively. Thereafter, the roots and shoots of these plants were separated. Fresh weights of shoots and roots were measured immediately after harvesting using an electronic balance. The plant materials were then dried in a ventilated oven at 70°C for 4–5 days until the dry weights were constant, and their dry weight was determined by an electronic balance.

### Substrate Properties

Substrate samples were air-dried, ground, and sieved through a 2-mm sieve to attain homogeneity in their properties.

The pH buffering capacity (pHBC) was determined using acid-base titration techniques (Xu et al., 2012).

Substrate samples were analyzed for TOC using the dry combustion method. OM was calculated as OM (g/kg) = TOC (g/kg) × 1.724 (Gao et al., 2018). CEC of the substrates was measured using the ammonium acetate compulsory displacement method (Xu et al., 2012).

The storage capacity of NH_4_^+^ of different substrates was measured by a modified method (Nieder et al., 2011). In detail, 1 g of substrate was weighed into each of three 50-mL polyethylene tubes, and 30 mL of different concentration NH_4_Cl solutions were added, whose concentrations were 0.001, 0.005, 0.01, 0.05, 0.1, 0.3, 0.5, and, 1 M (standardized), respectively. The suspensions were shaken for 24 h at 25°C and equilibrated for 1 day at 25°C, after that collected 10 mL of supernatant. The residual NH_4_^+^ in supernatants were determined by Kjeldahl procedure. Due to the addition amount of NH_4_^+^ was known, which could be calculated, combined with the data of the residual NH_4_^+^ in supernatants, and the total adsorption quantity of NH_4_^+^ of substrates treated by different concentration NH_4_Cl solutions were calculated. According to the above-mentioned data, the curves of NH_4_^+^ concentration and total adsorption quantity of NH_4_^+^ of treated substrates were fitted by Excel, absorption saturation point of different substrates were known. The treated substrates corresponding to the absorption saturation point were then dried at 50°C for 72 h prior to fixed NH_4_^+^ measurement. The method for the determination of fixed NH_4_^+^ is that 0.5 g of dried substrate was weighed into each of three 50 mL polyethylene tubes, and 20 mL of alkaline KOBr solution was added into each tube; the suspensions were shaken for 30 min and equilibrated for 2 h and then heated in a water bath and boiled for 5 min. After that, it was cooled and equilibrated the suspension overnight. The next day, the supernatants were discarded and the residues were washed by 40 mL of 0.5 M KCl three times, then 20 mL 5 N HF:1 N HCl was added into each tube and shaken for 24 h; all fixed NH_4_^+^ of substrates were released into the suspensions after the above procedure. Finally, the NH_4_^+^ released was determined by steam distillation of the substrate-sulfuric acid mixture after adding 1M NaOH by Kjeldahl procedure.

The storage capacity of P of different substrates was measured by a modified method (Butegwa et al., 1995). In detail, 1 g of substrate was weighed into each of three 50-mL polyethylene tubes, and 30 mL of different concentration KH_2_PO_4_ solutions were added, whose concentrations of P were 100, 300, 500, 1,000, 1,500, 2,000, 3,000, 4,000, 5,000, and 6,000 mg/L (standardized), respectively. The suspensions were shaken for 24 h at 25°C and equilibrated for 1 day at 25°C; after that were collected 10 mL of supernatant, the residual P in supernatants were determined by ICP-AES (ICP6300, Britain). Due to the additional amount of P being known, which could be calculated, combined with the data of residual P in supernatants, the total adsorption quantity of P of substrates treated by different concentration KH_2_PO_4_ solutions were calculated. According to the above-mentioned data, the curves of P concentration and total adsorption quantity of P of treated substrates was fitted by Excel, and absorption saturation point of different substrates were known. The treated substrates corresponding to the absorption saturation point were then dried at 50°C for 72 h prior to fixed phosphorus measurement. Exchangeable P was determined by extractions in 1% NH_4_HCO_3_ (pH 7.0) and analysis of the extracts with ICP-AES (ICP6300, Britain) following filtration through filter paper. Fixed P was calculated by subtracting the amount of exchangeable P from the total adsorption quantity of P.

The storage capacity of K^+^ of different substrates was measured by a modified method (Simonsson et al., 2009). In detail, 1 g of substrate was weighed into each of three 50 mL polyethylene tubes, and 30 mL of different concentration KCl solutions were added, whose concentrations of K^+^ were 100, 200, 400, 1,000, 1,500, 2,000, 3,000, 4,000, 6,000, and 8,000 mg/L (standardized), respectively. The suspensions were shaken for 24 h at 25°C and equilibrated for 1 day at 25°C; after that was collected 10 mL of supernatant, the residual K^+^ in supernatants was determined by ICP-AES (ICP6300, Britain). Due to the additional amount of K^+^ being known, which could be calculated, combined with the data of residual K^+^ in supernatants, the total adsorption quantity of K^+^ of substrates treated by different concentration KCl solutions was calculated. According to the above-mentioned data, the curves of K^+^ concentration and total adsorption quantity of K^+^ of treated substrates was fitted by Excel, and the absorption saturation point of different substrates were known. The treated substrates corresponding to the absorption saturation point underwent two additional wetting-drying cycles, which each involved shaking with 5 mL distilled water for 6 h and drying at 105°C for 16 h prior to fixed K^+^ measurement. Exchangeable K^+^ was determined by repeated extractions in 1 M NH_4_Ac (pH 7.0) and analysis of the extracts with ICP-AES (ICP6300, Britain) following centrifugation (2,000 rpm × 10 min) and filtration through a filter paper. Fixed K^+^ was calculated by subtracting the amount of exchangeable K^+^ from the total adsorption quantity of K^+^.

### Statistical Analysis

All data were analyzed using Excel 2019 and SPSS 17.0 software, and the statistical significance of the differences between treatments was determined by Duncan’s multiple range test (significance level *P* < 0.05).

## Results

### Morphological Parameters

There was a difference in plant height among the six treatments (Table 3). HA application increased the height of cucumber seedlings cultivated in pure cocopeat and the mixture of sand:cocopeat (1:1, v/v) by 17.59 and 20.02%, respectively, whereas the pure sand treatments showed an opposite trend when HA was applied, indicating that the three substrates responded differently to the application of HA. HA application to cocopeat or the mixture of sand:cocopeat (1:1, v/v) did not have effect on the stem diameter, whereas it decreased the stem diameter of cucumber seedlings grown in sand (by 16.29%) compared with that of cucumber seedlings grown in sand without HA addition. To investigate whether HA utilization affected cucumber biomass, we analyzed the fresh and dry weights of shoots and roots at the end of the trial period. Incorporation of 1% HA to the cocopeat increased the fresh and dry weight of roots by 26.37 and 17.86%, respectively, compared with that of roots of plants grown in cocopeat without HA application. HA addition to the mixture of sand:cocopeat (1:1, v/v) significantly increased the fresh and dry weight of shoots and roots compared with those of the groups without HA application. In contrast, there was a decrease in the fresh and dry weight of cucumber shoots and roots when 1% HA was applied to sand compared with that without HA addition.

**TABLE 3:** Effects of 1% HA addition on the morphological parameters of cucumber seedlings.

Treatments	Plant height (cm)	Stem diameter (mm)	Fresh weight of shoots (g)	Dry weight of shoots (g)	Fresh of roots (g)	Dry weight of roots (g)
T1	15.22 ± 1.01 e	3.50 ± 0.18 c	11.89 ± 0.42 d	2.52 ± 0.26 d	4.27 ± 0.13 e	0.88 ± 0.02 e
T2	12.33 ± 0.66 f	2.93 ± 0.29 d	9.59 ± 0.14 e	2.12 ± 0.07 e	2.35 ± 0.06 f	0.42 ± 0.07 f
T3	29.67 ± 1.45 b	4.31 ± 0.31 ab	18.77 ± 0.74 a	7.34 ± 0.92 a	7.62 ± 0.46 b	1.40 ± 0.18 b
T4	34.89 ± 2.71 a	4.77 ± 0.18 a	19.99 ± 1.68 a	7.70 ± 1.08 a	9.63 ± 0.39 a	1.65 ± 0.12 a
T5	18.33 ± 1.15 d	3.99 ± 0.19 b	14.16 ± 0.42 c	6.10 ± 0.48 c	6.41 ± 0.32 d	1.10 ± 0.04 d
T6	22.00 ± 0.33 c	4.31 ± 0.12 ab	16.14 ± 0.20 b	6.67 ± 0.31 b	6.88 ± 0.10 c	1.21 ± 0.05 c

*T1, sand; T2, sand + 1% HA; T3, cocopeat; T4, cocopeat + 1% HA; T5, 1:1 sand:cocopeat; T6, 1:1 sand:cocopeat + 1% HA. Data represents the average of three replicates ± SE. Different letters indicate significant differences at P < 0.05.*

### pH Buffering Capacity

**FIGURE 1:**
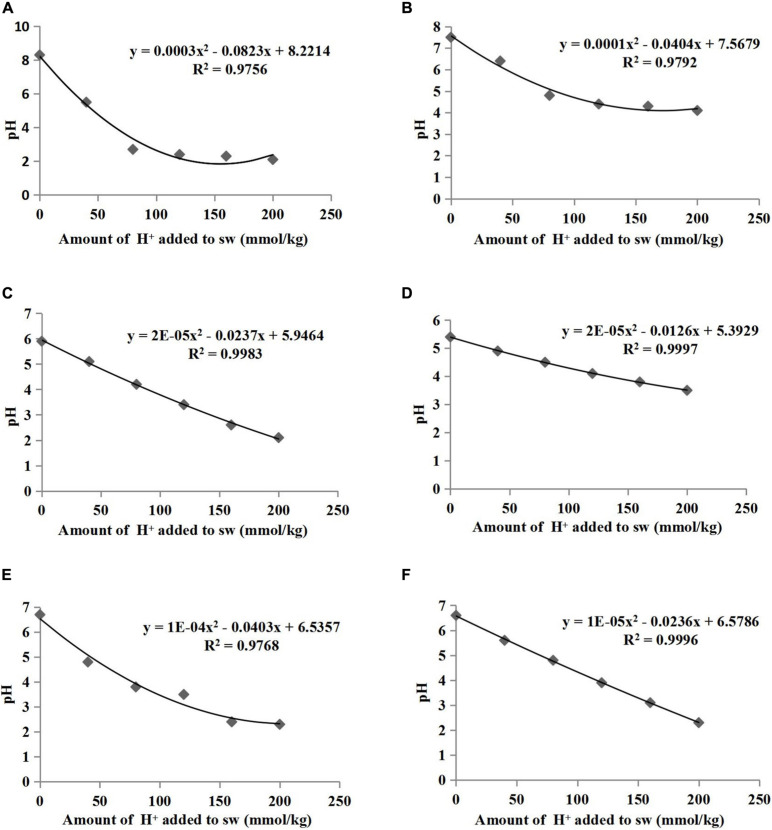
Relationships between the amount of acid added to substrate weight (sw) and the pH of sand **(A)**, sand+1% HA **(B)**, cocopeat **(C)**, cocopeat+1% HA **(D)**, 1:1 sand:cocopeat **(E)**, and 1:1 sand:cocopeat +1% HA **(F)** suspensions.

**FIGURE 2:**
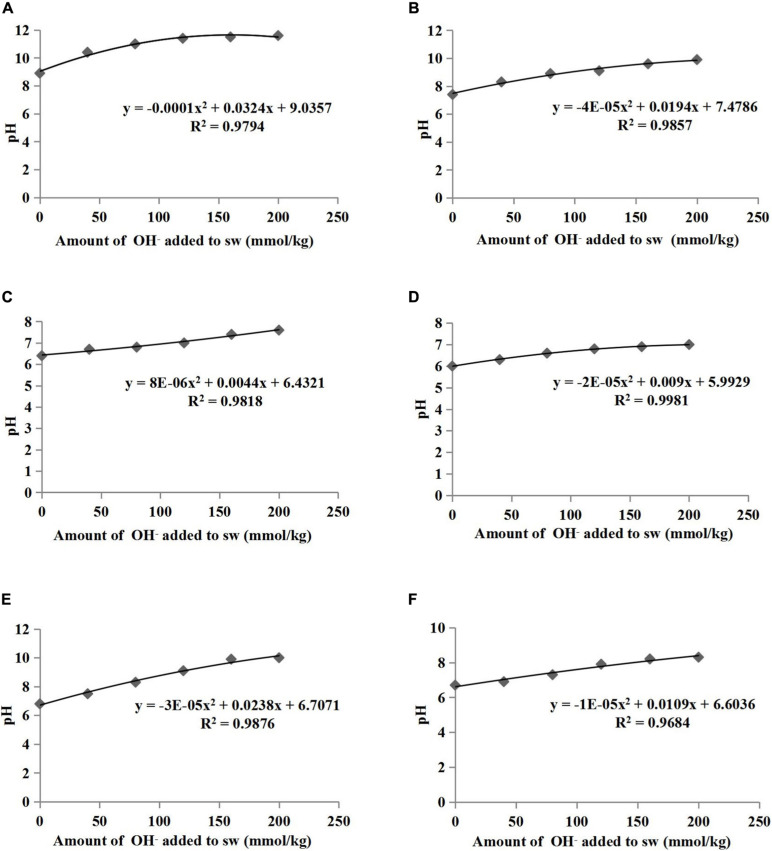
Relationships between the amount of alkali added to substrate weight (sw) and the pH of sand **(A)**, sand+1% HA **(B)**, cocopeat **(C)**, cocopeat+1% HA **(D)**, 1:1 sand:cocopeat **(E)**, and 1:1 sand:cocopeat +1% HA **(F)** suspensions.

**FIGURE 3:**
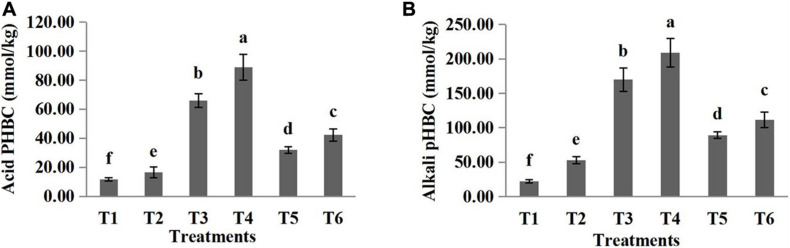
Acid **(A)** and alkali **(B)** buffering capacity of different substrates. Bars represent standard errors. T1, sand; T2, sand + 1% HA; T3, cocopeat; T4, cocopeat + 1% HA; T5, 1:1 sand:cocopeat; T6, 1:1 sand:cocopeat + 1% HA. Different letters above the bars indicate significant differences at *P* < 0.05 according to Duncan’s multiple range test.

The pH buffering capacity (pHBC) was defined as the number of moles of H^+^ or OH^–^ necessary to increase and decrease the pH of 1 kg of soil by 1 pH unit (Mowbray and Schlesinger, 1988). The acid and alkali buffering curves for the six treatments are presented in Figures 1, 2, respectively, and all correlation coefficients of the quadratic regression curve (*R*^2^) were > 0.95. The pHBC values for the six treatments were calculated using the corresponding regression equations of the buffering curves. In the case when the same amount of acid was added, the faster the pH value decreased, the lower the acid buffering capacity of the substrate, whereas in the case when the same amount of alkali was added, the faster the pH value increased, the lower the alkali buffering capacity of the substrate. The substrates with poor acid or alkali buffering capacity showed a more dramatic change in the acid or alkali buffering curve. As shown in Figures 1, 2, the pHBC of the groups without HA addition fluctuated more sharply after acid or alkali addition than that of the groups to which HA was applied. As shown in Figure 3, there was a great variation in pHBC among the different groups. The presence of HA contributed to an increase in acid and alkali pHBC compared with those in groups without HA application. In addition, among the three types of substrates, the greatest acid and alkali pHBC were observed in the cocopeat groups, whereas the groups with the mixture of sand:cocopeat (1:1, v/v) showed better performance of acid and alkali pHBC than that of the sand groups.

### TOC, OM, and CEC

As shown in Table 4, higher TOC and OM were observed in the substrates with 1% HA application than those in the substrates without HA application, and CEC was consistently influenced by HA application. Moreover, in comparison to sand and the mixture of sand:cocopeat (1:1, v/v) treatments, the cocopeat treatment performed better with respect to TOC, OM, and CEC.

**TABLE 4:** Effects of 1% HA addition on total organic carbon (TOC), organic matter (OM), and cation exchange capacity (CEC) of different substrates.

Treatments	TOC (g/kg)	OM (g/kg)	CEC (cmol/kg)
T1	2.40 ± 0.60 f	4.14 ± 1.03 f	1.37 ± 0.29 f
T2	10.20 ± 0.60 e	17.58 ± 1.03 e	4.77 ± 0.25 e
T3	67.20 ± 1.59 b	115.85 ± 2.74 b	17.33 ± 0.86 b
T4	76.80 ± 1.59 a	131.89 ± 2.74 a	27.43 ± 0.58 a
T5	21.40 ± 1.83 d	36.89 ± 3.16 d	9.37 ± 0.76 d
T6	41.80 ± 0.92 c	72.06 ± 1.58 c	12.47 ± 0.46 c

*T1, sand; T2, sand + 1% HA; T3, cocopeat; T4, cocopeat + 1% HA; T5, 1:1 sand:cocopeat; T6, 1:1 sand:cocopeat + 1% HA. Data represents the average of three replicates ± SE. Different letters indicate significant differences at P < 0.05.*

### Storage Capacity of NH_4_^+^ in Different Substrates

**FIGURE 4:**
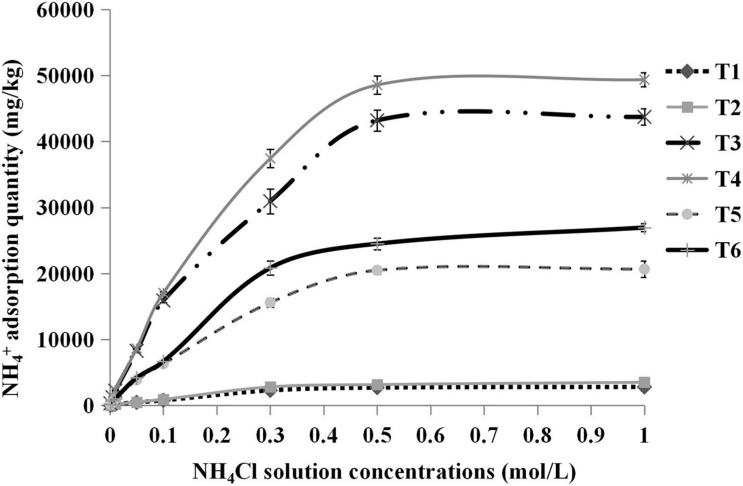
Relationships between the concentrations of NH_4_Cl solution added and the total NH_4_^+^ adsorption quantity. T1, sand; T2, sand + 1% HA; T3, cocopeat; T4, cocopeat + 1% HA; T5, 1:1 sand:cocopeat; T6, 1:1 sand:cocopeat + 1% HA.

As shown in Figure 4, it could be clearly noted that the adsorption saturation points varied among the substrates. In the case of pure sand and pure cocopeat groups, total NH_4_^+^ absorption tended to be saturated when the concentration of NH_4_Cl solution reached 0.3 and 0.5 M, respectively. The total NH_4_^+^ adsorption curves of all treatments could be divided into two distinct parts. In the first part, NH_4_Cl solution concentrations were below the saturation point, and the total NH_4_^+^ adsorption quantity increased greatly with increasing solution concentrations. The second part of the curve was associated with the NH_4_Cl solution concentrations beyond the saturation point, at which all absorption curves became relatively flat, which indicated that they were almost saturated. Furthermore, all substrates were already saturated with 1.0 M NH_4_Cl solution. In relation to the abovementioned results, we calculated the storage capacity of NH_4_^+^ at the complete saturation point (the corresponding concentration of NH_4_Cl solution was 1.0 M). The storage capacity of NH_4_^+^ was related to three parameters, including total NH_4_^+^, fixed NH_4_^+^, and exchangeable NH_4_^+^ adsorption quantities. Among the three types of substrates, the storage capacity of NH_4_^+^ in cocopeat and in sand were the highest and the lowest, respectively. As shown in Table 5, HA application seemed to be an important factor that influenced the NH_4_^+^ adsorption of substrates due to the significantly higher total NH_4_^+^, fixed NH_4_^+^, and exchangeable NH_4_^+^ adsorption in the substrates to which 1% HA was applied. Additionally, most of the adsorbed NH_4_^+^ was present in the exchangeable form, which could easily be released from substrates and used by crops when needed.

**TABLE 5:** Effects of 1% HA addition on NH_4_^+^ storage capacity of different substrates.

Treatments	Total NH_4_^+^ adsorption quantity (mg/kg)	Exchangeable NH_4_^+^ adsorption quantity (mg/kg)	Fixed NH_4_^+^ adsorption quantity(mg/kg)
T1	2800.00 ± 396.76 f	2798.94 ± 306.15 f	1.07 ± 0.12 f
T2	3500.00 ± 401.22 e	3498.76 ± 298.11 e	1.64 ± 0.11 e
T3	43715.00 ± 1243.85 b	43702.46 ± 1243.69 b	12.54 ± 0.51 b
T4	49350.00 ± 1050.00 a	49335.45 ± 1049.78 a	16.55 ± 0.43 a
T5	20650.00 ± 1212.44 d	20643.91 ± 1212.84 d	6.09 ± 0.43 d
T6	26950.00 ± 613.25 c	26942.23 ± 606.36 c	7.77 ± 0.18 c

*T1, sand; T2, sand + 1% HA; T3, cocopeat; T4, cocopeat + 1% HA; T5, 1:1 sand:cocopeat; T6, 1:1 sand:cocopeat + 1% HA. Data represents the average of three replicates ± SE. Different letters indicate significant differences at P < 0.05.*

### Storage Capacity of P in Different Substrates

**FIGURE 5:**
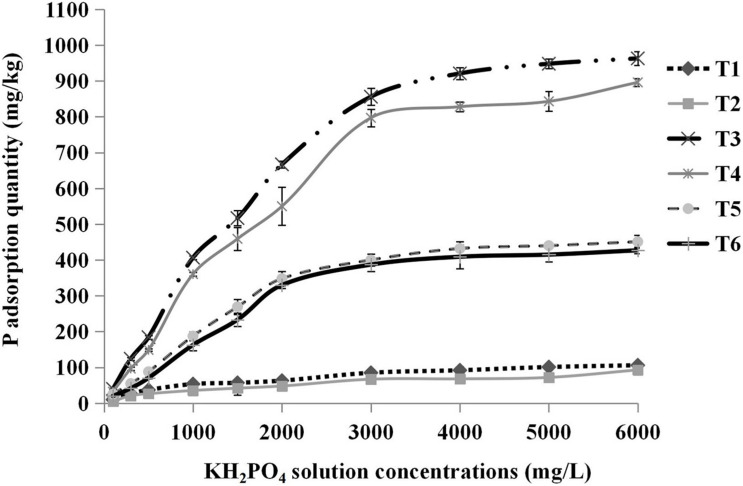
Relationships between the concentrations of KH_2_PO_4_ solution added and the total P adsorption quantity. T1, sand; T2, sand + 1% HA; T3, cocopeat; T4, cocopeat + 1% HA; T5, 1:1 sand: cocopeat; T6, 1:1 sand: cocopeat + 1% HA.

The relationships between the concentrations of KH_2_PO_4_ solution added and the total phosphorus adsorption quantities of different substrates are shown in Figure 5. The total P adsorption quantity increased with increasing solution concentrations until the substrates were completely saturated with P. The absorption saturation points of pure sand, pure cocopeat, and the mixture of sand:cocopeat (1:1, v/v) were approximately 2,000, 3,000, and 2,000 mg/L, respectively. To ensure that all substrates were in a state of complete saturation and to obtain accurate results, we selected the 6,000 mg/L point to calculate the storage capacity of P for different types of substrates, and the corresponding results are presented in Table 6. Cocopeat treatments absorbed the greatest amount of P, whereas sand treatments absorbed the least, which was in accordance with the results of the NH_4_^+^ absorption measurement. According to the analysis of the variance, no significant difference in exchangeable P was detected between HA application and no HA application treatments, whereas the treatments without HA addition were higher than those with HA addition on both total and fixed P adsorption quantity for three types of substrates.

**TABLE 6:** Effects of 1% HA addition on phosphorus storage capacity of different substrates.

Treatments	Total P adsorption quantity (mg/kg)	Exchangeable P adsorption quantity (mg/kg)	Fixed P adsorption quantity (mg/kg)
T1	106.00 ± 4.58 e	89.90 ± 5.25 c	16.10 ± 1.91 c
T2	93.00 ± 3.00 f	86.03 ± 2.72 c	6.97 ± 0.39 d
T3	953.00 ± 12.12 a	889.71 ± 15.37 a	63.29 ± 8.71 a
T4	896.00 ± 10.54 b	874.04 ± 13.35 a	21.96 ± 3.88 b
T5	451.00 ± 8.08 c	424.67 ± 16.71 b	26.33 ± 4.98 b
T6	427.00 ± 9.17 d	412.24 ± 10.48 b	14.76 ± 2.39 c

*T1, sand; T2, sand + 1% HA; T3, cocopeat; T4, cocopeat + 1% HA; T5, 1:1 sand: cocopeat; T6, 1:1 sand: cocopeat + 1% HA. Data represents the average of three replicates ± SE. Different letters indicate significant difference at P < 0.05.*

### Storage Capacity of K^+^ in Different Substrates

**FIGURE 6:**
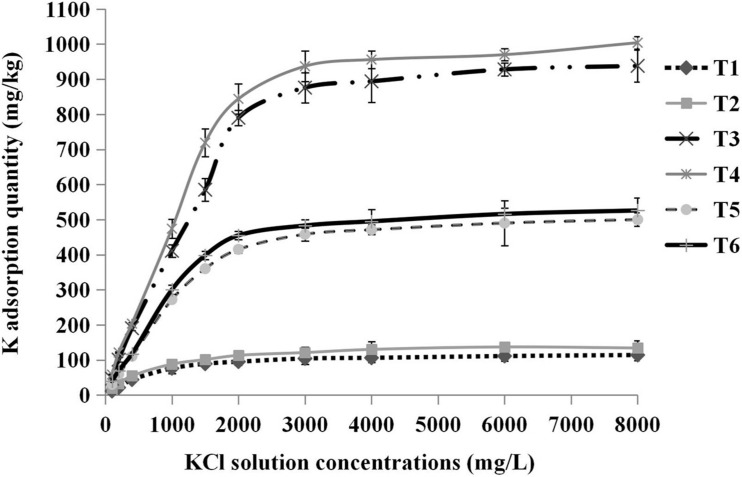
Relationships between the concentrations of KCl solution added and the total K adsorption quantity. T1, sand; T2, sand + 1% HA; T3, cocopeat; T4, cocopeat + 1% HA; T5, 1:1 sand: cocopeat; T6, 1:1 sand: cocopeat + 1% HA.

We found that there was a difference in the K adsorption saturation point among different substrates. Pure sand, pure cocopeat, and the mixture of both were nearly saturated when the concentration of KCl solution was 2,000, 3,000, and 2,000 mg/L, respectively. The shapes of the total K adsorption curves were similar to those of the NH_4_^+^ adsorption curves, and the substrates were completely saturated when they were treated with the 8,000 mg/L KCl solution. Based on these results, we calculated the storage capacity of K at the saturation point (the concentration of the corresponding KCl solution was 8,000 mg/L). The storage capacity parameters of K included total K, fixed K, and exchangeable K adsorption quantities. It was apparent that the storage capacity parameters of K in cocopeat were the highest among the three substrate types (Figure 6). As shown in Table 7, for the three types of substrates, the total K, fixed K, and exchangeable K adsorption quantities were enhanced by HA addition compared with those without HA addition. Moreover, we also observed that exchangeable K played a key role in the amount of total adsorbed K, and this was consistent with the results of NH_4_^+^ absorption.

**TABLE 7:** Effects of 1% HA addition on potassium storage capacity of different substrates.

Treatments	Total K^+^ adsorption quantity (mg/kg)	Exchangeable K^+^ adsorption quantity (mg/kg)	Fixed K^+^ adsorption quantity (mg/kg)
T1	104.00 ± 6.39 f	81.67 ± 5.80 f	22.33 ± 2.31 d
T2	132.00 ± 9.17 e	103.87 ± 7.41 e	28.13 ± 2.92 c
T3	938.00 ± 25.43 b	698.53 ± 37.04 b	239.47 ± 21.37 a
T4	1004.00 ± 18.33 a	782.93 ± 15.03 a	221.07 ± 33.35 a
T5	500.00 ± 9.29 d	397.48 ± 15.90 d	102.52 ± 12.57 b
T6	526.00 ± 9.17 c	426.59 ± 4.81 c	99.41 ± 11.38 b

*T1, sand; T2, sand + 1% HA; T3, cocopeat; T4, cocopeat + 1% HA; T5, 1:1 sand: cocopeat; T6, 1:1 sand: cocopeat + 1% HA. Data represents the average of three replicates ± SE. Different letters indicate significant difference at P < 0.05.*

## Discussion

### Effects of HA Addition to Different Substrates on Plant Morphological Parameters

We comparatively evaluated the effects of HA addition on three differently textured substrates and found that the effects varied. The results revealed that the addition of 1% HA to the pure cocopeat substrate and to a mixture of sand:cocopeat (1:1, v/v) had a positive effect on the morphological characteristics of cucumber plants, which was consistent with previous results related to soil measurements (Dobbss et al., 2007). The reason for this may be that HA benefits various biochemical processes. Several studies indicate that HA has auxin-like activity, stimulating root development through a significant proliferation of lateral roots and activation of H^+^-ATPases and H^+^-PPase in vacuoles as well as in the plasmalemma (Zandonadi et al., 2007). Roots are the main plant organ for the absorption of nutrients and water, and an optimal root system is one of the requirements for yield formation (Liu et al., 2015). In the present study, the root biomass of cucumber seedlings with HA addition was higher than that of cucumber seedlings without HA addition in the pure cocopeat and in the mixture of sand:cocopeat (1:1, v/v) treatments. In addition, HA can also increase the water-holding capacity of the substrate to alleviate water stress, which may explain why HA benefits plants (García et al., 2014). HA addition achieved favorable results in pure cocopeat and in the mixture of sand:cocopeat (1:1, v/v), whereas the results were completely opposite in the pure sand groups. This may be attributed to the poor acid pHBC of sand as shown in Figure 3A. There were differences in acid pHBC between sand and the other substrates. HA is an acidic substance, and pH is one of the most representative indicators of substrate chemical properties (Gao et al., 2018). When HA was added to sand, pH decreased dramatically, owing to the poor acidic pHBC, and improper rhizospheric pH led to physiological disorder of roots, thereby severely affecting plant uptake of water and nutrients, which consequently resulted in low plant biomass (Wang et al., 2016).

Morphological plant parameters can indirectly reflect substrate quality. In the present study, all morphological parameters of cucumber seedlings differed among the three substrates, and seedlings in the cocopeat groups showed the best performance. Previous studies demonstrate that cocopeat is suitable for use as a growing substrate because of its excellent physical properties, such as high water-holding capacity and porosity, which are beneficial to root development (Zhu et al., 2019). In addition, the root system releases some CO_2_ during root respiration that must dissipate from the root environment (Li et al., 2018), and because cocopeat has high porosity, CO_2_ could rapidly dissipate from the rhizosphere. Compared with cocopeat, sand was more compact and had a lower water retention capacity, which led to a severe restriction of root growth (Liu et al., 2015). Therefore, significant differences were observed in the morphological parameters of plants grown in pure cocopeat and those grown in pure sand.

### Effects of HA Addition to Different Substrates on pHBC, TOC, OM, and CEC

Soil pHBC refers to the ability of soil to mitigate the change in pH after the addition of acidic or alkaline substances, and it includes acid and alkali buffering capacity (Lieb et al., 2011). In general, the application of chemical fertilizers greatly affects soil pH; for instance, NH_4_^+^ application results in acidification because plants absorb more cations than anions and consequently release protons to the soil, whereas excess nitrates lead to soil alkalization (Schaller, 1987). Logically, soils with low pHBC are sensitive to acidification or alkalization, which greatly influences their nutrient availability and thereby the acquisition of nutrients by plants (Shi et al., 2017). Therefore, substrates with high pHBC are beneficial for cultivation. In the present study, the incorporation of HA resulted in stronger pH buffering behavior of the three substrates (Figure 3), indicating that HA addition is beneficial for stabilizing the rhizosphere pH, and consequently promoting plant growth.

As shown in Table 4, compared with the treatments without HA addition, the treatments with HA addition increased TOC and OM in the three different substrates. HA is a macromolecular organic substance rich in organic carbon (Saidimoradi et al., 2019), which is why mixing HA into substrates enhanced TOC and OM in the three substrates (Table 4). A previous study reported that the content of OM in the soil was positively correlated with soil pHBC (Xu et al., 2012). This is because there is an ample amount of oxygen-containing functional groups, such as carboxyl and phenolic hydroxyl groups, on the surface of OM (Liang et al., 2011). These groups accept protons when pH decreases and provide protons when pH increases (Kida et al., 2005), making a great contribution to pHBC improvement. Furthermore, we found that in the HA application treatments, the alkali buffering capacity of the three substrates was greater than their acid buffering capacity. The reason for this was because HA carries abundant acidic functional groups which can effectively neutralize alkaline components.

Cation exchange capacity (CEC) is a crucial index for assessing substrate quality, and it describes the potential of a substrate to absorb and release nutrients. High CEC can reduce fluctuations in ion concentrations (Pincus et al., 2017). As shown in Table 4, there was a difference in CEC between the treatments with and without HA addition. Previous studies have demonstrated that there was a positive correlation between CEC and OM. The reason for this positive correlation is that OM possesses a large number of functional groups which can be hydrolyzed to produce negative charges; these negative charges are able to adsorb a high amount of exchangeable cations and consequently improve CEC (Shekofteh et al., 2017).

Our results show that the TOC and OM of cocopeat groups were higher than those of the other groups (Table 4). Cocopeat is an agricultural by-product manufactured after the selection of fiber from coconut husks (Hongpakdee and Ruamrungsri, 2017); thus, it is rich in OM. On the other hand, sand is an inorganic substrate; therefore, TOC and OM differed significantly among the three substrates. In addition, it could be clearly observed that pHBC of cocopeat was significantly higher than that of the other two substrates (Figure 3), which was consistent with the high OM content of cocopeat. Furthermore, the CEC results coincided with OM measurements as shown in Table 4. The CEC of the cocopeat was significantly higher than that of the other two substrates. Additionally, in the mixture of sand:cocopeat (1:1, v/v), cocopeat addition compensated for the deficiency of OM in sand and consequently improved the CEC.

### Effects of HA Addition to Different Substrates on N, P, and K Storage Capacities

The results show that the presence of HA increased the NH_4_^+^ storage capacity in the three different substrates (Table 5). CEC affects the soil storage capacity for nutrients (Wang et al., 2019); therefore, a difference in CEC between HA groups and no HA groups might lead to differences in NH_4_^+^ storage capacity. Additionally, the chemical reaction of NH_4_^+^ with HA is closely linked to NH_4_^+^ storage because these two substances frequently form NH_4_^+^ humate, which has higher stability than other NH_4_^+^ salts. NH_4_^+^ volatilization loss was reduced by this process, and this ultimately increased the storage capacity of NH_4_^+^ (Dong et al., 2009). Furthermore, NH_4_^+^ could be incorporated into HA through covalent bonding, and the majority of NH_4_^+^ appears to be in the form of indole and pyrrole N (Thorn and Mikita, 1992). This may also explain why HA addition actively modified NH_4_^+^ storage capacity. Moreover, a previous study showed that HA could easily bind with NH_4_^+^ owing to its strong complexation and absorption capabilities. In the present study, HA showed significant slow-release effects on N release and utilization, which consequently increased fertilizer use efficiency (Chen et al., 2017). Fixed NH_4_^+^ and exchangeable NH_4_^+^ are the main constituents of adsorbed NH_4_^+^ in the soil (Egashira et al., 1998). Exchangeable NH_4_^+^ can be directly used by plants (Beuters et al., 2014), and this accounted for the majority of absorbed NH_4_^+^ in the present study (Table 5), implying that the adsorption of NH_4_^+^ by the three substrates was temporary and easily taken up by plants. Compared with that in the groups without HA addition, higher exchangeable NH_4_^+^ was observed in the HA addition groups, indicating that HA could improve the exchangeable NH_4_^+^ storage capacity in the three substrates.

P is an essential nutrient for plant growth, and phosphate deficiency can disrupt plant metabolism (Yang et al., 2004); therefore, the availability of phosphates is an important agricultural issue. Once phosphate fertilizers are applied to soils, a series of complex reactions that decrease phosphate availability may gradually occur. The main reactions include phosphate adsorption into soil and precipitation in the form of calcium phosphate minerals (Parfitt et al., 2010; Perassi and Borgnino, 2014), which can adversely affect the uptake of phosphorus by plant roots. In our experiment, the results showed that HA addition decreased total P adsorption, indicating that it has a desirable impact on the improvement of phosphate availability. It is generally accepted that HA is rich in anions, such as hydroxyl and carboxyl ions (Liang et al., 2011), and that it can reduce the adsorption of soil minerals to phosphates because of the strong competition of both anions for the same adsorption sites on the surface of soil minerals. In addition, HA can bind with Ca^2+^ in the soil and interact with Ca-P to inhibit the formation of Ca-P precipitates. These effects improved the mobility of P and ultimately enhanced P availability in the soil (Cimrin and Yilmaz, 2005; Antelo et al., 2007; Perassi and Borgnino, 2014; Zhou et al., 2015). The results of our study were in accordance with those of previous studies as shown in Table 6. HA addition could suppress phosphate fixation in the three different substrates; in other words, the availability of phosphates was increased.

The release of absorbed K^+^ in the soil occurred in the rhizosphere when K^+^ concentration in the rhizosphere solution decreased below the threshold (Hinsinger and Jaillard, 2010). HA exerts a regulatory effect on nutrient forms in fertilizers, and it could react with K^+^ in fertilizers to form K^+^ humate, which is not easily lost with water, and the presence of potassium humate can provide the ability to slowly release potassium (Liu et al., 2006). This means that HA application directly enhances the storage capacity of K^+^ in the soil and modifies the potassium supply pattern. Our results are in accordance with the results of earlier research as shown in Table 7. The total K^+^ adsorption of the three different substrates with HA application was significantly higher than that of the three groups without HA application. The total adsorbed K^+^ comprises exchangeable K^+^ and fixed K^+^ (Askegaard et al., 2003). In the present study, exchangeable K^+^ accounted for the majority of adsorbed K^+^. Exchangeable K^+^ in the rhizosphere serves as the main source of K for plants (Madaras and Koubová, 2016). In our experiment, the amount of absorbed exchangeable K^+^ in the HA addition groups was significantly higher than that in the groups without HA addition, and the difference in this indicator might explain the difference in plant growth between the treatments with and without HA addition. Furthermore, a previous study demonstrated that HA could enhance the proportion of exchangeable K^+^ in adsorbed K^+^ (Olk and Cassman, 1995). In the present study, the presence of HA increased the proportion of exchangeable K^+^ in adsorbed K^+^.

## Conclusion

Incorporation of HA to different texture substrates had different effects on plant growth parameters (plant height, stem diameter, and biomass). In pure cocopeat and the mixture of sand: cocopeat (1:1, v/v), HA application improved the growth of cucumber seedlings, whereas in sand, HA addition was harmful to plant growth. The comprehensive evaluation showed that pHBC, organic matter content, and CEC in the HA groups were higher than those in the groups without HA. Treatments with HA addition resulted in higher NH_4_^+^ and K^+^ storage capacity, while decreasing P fixation, thus increasing P availability, indicating that HA application enhanced fertilizer efficiency. Among the three substrates, cocopeat was the best substrate for cultivation, and incorporating cocopeat into sand was beneficial to plant growth. Overall, cocopeat with 1% HA was the best treatment in this study.

## Data Availability Statement

The original contributions presented in the study are included in the article/Supplementary Material, further inquiries can be directed to the corresponding author/s.

## Author Contributions

WJ, HY, QL, and TL contributed to the conception and design of the study. JX and EM conducted the experiments, collected the data, and performed the statistical analysis. JX wrote the first draft of the manuscript. WJ, HY, and TL contributed to the writing and revision of the manuscript. All authors have given final approval for the publication of the manuscript.

## Conflict of Interest

The authors declare that the research was conducted in the absence of any commercial or financial relationships that could be construed as a potential conflict of interest.

## Publisher’s Note

All claims expressed in this article are solely those of the authors and do not necessarily represent those of their affiliated organizations, or those of the publisher, the editors and the reviewers. Any product that may be evaluated in this article, or claim that may be made by its manufacturer, is not guaranteed or endorsed by the publisher.

## Abbreviations

CEC: Cation exchange capacity
HA: humic acid
OM: Organic carbon
pHBC: pH buffering capacity
TOC: Total organic carbon.
