# Supplementary figures and images for: Effect of Humic Acid Addition on Buffering Capacity and Nutrient Storage Capacity of Soilless Substrates
Source: Front Plant Sci. 2021 Jul 26;12:644229. doi: 10.3389/fpls.2021.644229 (PMC8350343; doi:10.3389/fpls.2021.644229)

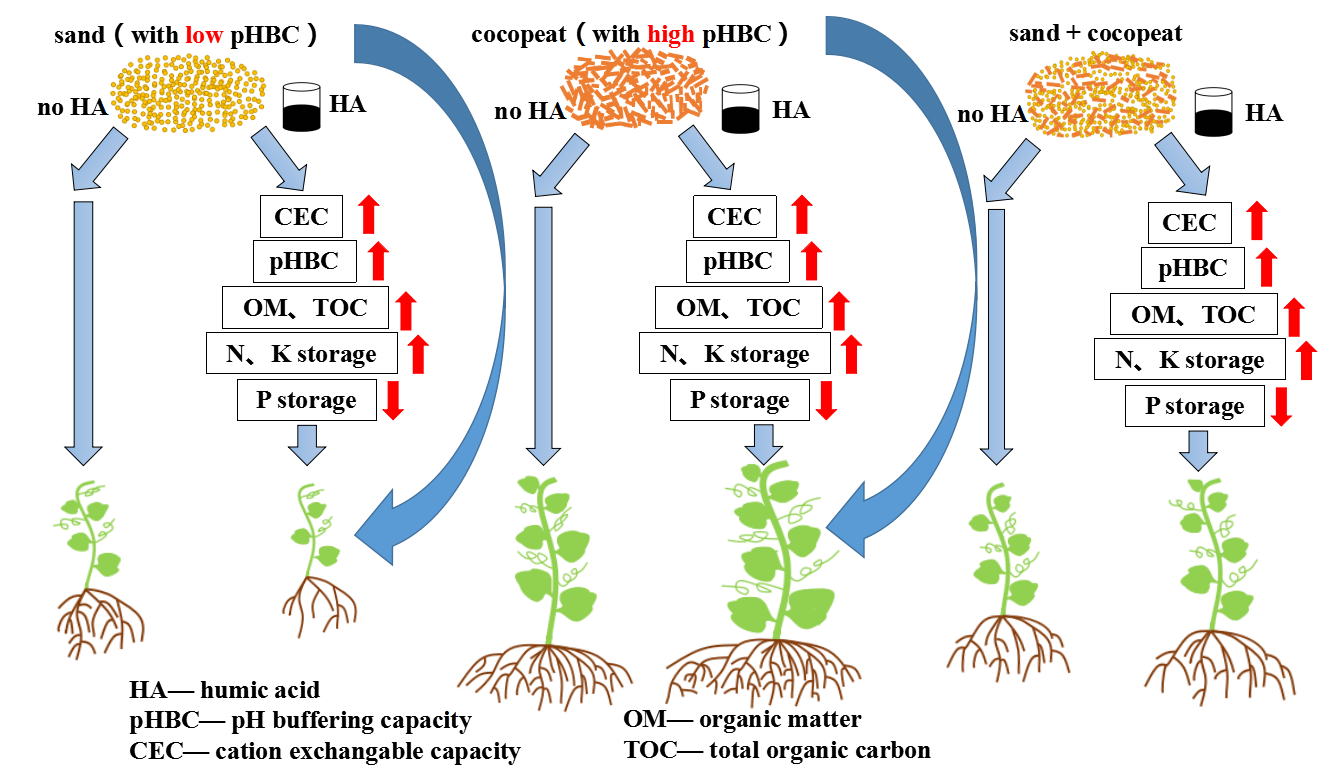

Supplement: Supplementary file 1 [file Image_1.TIF]
